# Climate legacy in seed and seedling traits of European beech populations

**DOI:** 10.3389/fpls.2024.1355328

**Published:** 2024-06-07

**Authors:** Tomasz A. Pawłowski, Jan Suszka, Joanna Mucha, Marcin Zadworny, Shirin Alipour, Barbara Kurpisz, Paweł Chmielarz, Andrzej M. Jagodziński, Daniel J. Chmura

**Affiliations:** ^1^ Institute of Dendrology, Polish Academy of Sciences, Kórnik, Poland; ^2^ Faculty of Forestry and Wood Technology, Poznan University of Life Sciences, Poznań, Poland

**Keywords:** adaptation, biodiversity, climate change, conservation, phenotypic variability, regeneration, reproduction, seed germination

## Abstract

Tree species’ ability to persist within their current distribution ranges is determined by seed germination and seedling growth. Exploring variation in these traits in relation to climatic conditions helps to understand and predict tree population dynamics, and to support species management and conservation under future climate. We analyzed seeds and seedlings of 26 European beech populations from the northeastern boundary of the species range to test whether: 1) adaptation to climatic conditions is reflected in depth of dormancy and germination of seeds; 2) climatic characteristics of origin predictably affect seedling traits. The variation in seed dormancy and germination in a laboratory test, and seedling growth and morphology traits in a nursery common-garden test was examined. Populations originating from warmer and drier sites (mostly from the northern region), compared to those from the opposite end of climatic gradient, germinated later, with a lower success, and produced seedlings with shorter and tougher roots. They had deeper dormancy and poorer seed germination capacity, and are likely more vulnerable to environmental changes. The climatic conditions at the origin shape the intraspecific variation of seed germination and seedling traits, and may limit regeneration from seed and affect adaptation potential of beech to increasing temperatures and decreasing precipitation.

## 1 Introduction

Changing environmental conditions might alter the demographic dynamics of populations and species distribution ranges (Iverson et al., 2004; Dyderski et al., 2018). At the northern edges of tree species ranges in Europe the increase in temperature is expected to broaden the suitable niche for some species (Dyderski et al., 2018; Chakraborty et al., 2021), however, direct genetic adaptations to the specific local habitats may reduce such expectancy. Environmental changes may cause disturbances in tree regeneration if future climatic conditions do not match the historically imprinted adaptation requirements for seed germination and seedling fitness (Eggers et al., 2008; Walck et al., 2011; Nadeau and Urban, 2019; Klupczyńska and Pawłowski, 2021). As various tree species may be differentially affected by those changes, the effects will be reflected in the composition and structure of future forests.

According to the concept of the regeneration niche, being one of the components of the plant ecological niche, the requirements are defined for the successful reproduction, dispersal, germination and seedling establishment (Grubb, 1977). During the last decade, this concept has received considerable research attention, as it helps to better understand species distributions, community assembly, population dynamics, and plant responses to environmental changes (Rosbakh et al., 2018). The ecological niche of tree species is typically narrower at earlier than at later life stages, and intraspecific genetic variation of early fitness traits may define response of tree populations to changing environmental conditions (Jackson et al., 2009; Donohue et al., 2010; Walck et al., 2011; Solé-Medina et al., 2020). To assess the adaptive potential, there is a need to recognize intraspecific genetic variation in fitness-related traits, and its environmental determinants (Alberto et al., 2013; Kurpisz and Pawłowski, 2022). Seed dormancy breaking and the timing of germination have important implications for the success of plant establishment in the face of environmental risk factors (Pausas et al., 2022; Zhang et al., 2022). However, the role of seed dormancy and germination in the success of trees remains underestimated (Fletcher et al., 2020). Thus, improving our understanding of intraspecific genetic variation in early fitness traits, such as seed dormancy, germination, seedling establishment and seedling shoot and root characteristics, is essential to elucidate the potential ability of tree populations to regenerate and persist under climate change (Solé-Medina et al., 2020).

European beech (*Fagus sylvatica* L.) is a sub-Atlantic tree species, and an important component of forest communities in Western and Central Europe (Bohn et al., 2000). The natural distribution range corresponds largely to typically temperate climate. It extends from southern Norway and Sweden in the north to Sicily in the south and from southern England, Brittany and the Cantabrian Mountains in the west to north-eastern Poland, the Carpathian Mountains and the Crimean Peninsula in the east, and it reaches the Balkans, the Rhodopes and Pindos Mountains in south-eastern Europe (Bohn et al., 2000). Beech arrived at the Baltic coast between 1500 and 1000 BC and now represents descendants of the Western and Central (in northern Poland) and Southern (in southern Poland) European refugia (Bohn et al., 2000; Leuschner and Ellenberg, 2017; Stefanini et al., 2022; Willner et al., 2023). Beech forests have mainly developed by natural regeneration, and ecotypes have developed that are adapted to the local climatic conditions (Bolte et al., 2007; Leuschner and Ellenberg, 2017). It shows the ecotypic nature of variation in growth traits (Barzdajn, 2009; Mátyás et al., 2009; Gömöry and Paule, 2011; Robson et al., 2013), whereas clinal variation was found for spring phenology (Wuehlisch et al., 1995; Chmura and Rożkowski, 2002; Robson et al., 2013). However the plasticity in response of beech populations to local conditions has also been discussed (Kramer et al., 2017), and in particular the plasticity of seed dormancy and germination has not been sufficiently researched. Seeds ripen and are released in late autumn, during winter they lose dormancy and in spring next year they germinate when conditions are favorable for seedling establishment.

Beech is known to be drought-sensitive (Kramer et al., 2008), thus its populations at the southern limit of the species distribution are the most endangered by climate change (Jump et al., 2006; Benito-Garzón et al., 2013; Robson et al., 2013). However, the northeastern border of its natural range is limited by both cold temperatures and drought (Bolte et al., 2007; Kreyling et al., 2014; Matisons et al., 2017; Farahat and Linderholm, 2018). There is a growing concern about the future survival and sustainability of beech ecosystems throughout Europe (Geßler et al., 2007; Aranda et al., 2015; Jastrzębowski et al., 2017), as beech may be unable to adapt to future environmental conditions in its current natural range (Lakatos and Molnár, 2009; Bellard et al., 2012; Stojnić et al., 2018). Protection of genetic diversity is essential both for economic reasons and to guarantee the adaptability of the common beech towards climatic changes (Bohn et al., 2000; Staszak and Pawłowski, 2012; Staszak et al., 2019). Populations growing at marginal sites are generally subject to unsuitable conditions and have a lower chance of survival (Kreyling et al., 2014). Marginal populations may be more resistant to climate disturbances than populations from more central areas in Europe if enough genetic variation is present within them (Sagarin and Gaines, 2002; Rose et al., 2009; Thiel et al., 2014). Due to the evolutionary processes to which they have been subjected, they may constitute important gene pools (Hampe and Petit, 2005) better adapted to future climate conditions (Stojnić et al., 2018). The future climatic conditions in parts of the distribution center and at the rear (southern) edge might thus become limiting for natural beech regeneration by seed, as the likelihood of extreme heat and drought events will increase (Nussbaumer et al., 2020; Muffler et al., 2021). At the leading (northern) edge, the increasing temperatures may increase germination success and beech regeneration, but more detailed studies are needed to elucidate the effect of climatic warming on beech regeneration (Muffler et al., 2021).

The aim of this study was to determine intraspecific genetic variability among the north-eastern European beech populations in early fitness traits - seed dormancy and germination in relation to the climate at population origin. The early growth of seedlings was also investigated in a nursery common-garden experiment to define how the climate of seed origin affects the fitness of seedlings. We expected that the results would allow recognition of the climatic factors related to the ability of individual beech populations to cope with and adapt to climate variability. This understanding is crucial for the assessment of the future of beech forests in Europe. We hypothesized that 1) variation in dormancy and germination of beech seeds is associated with climatic conditions acting on the populations; 2) climatic characteristics of seed provenances affect seedling traits. We expected that depth of dormancy of beech populations will increase from north to south within the analyzed geographic region in accordance with temperature gradient. We also expected that seed germination success and seedlings growth will be better in populations from the sites with milder climate.

## 2 Material and methods

### 2.1 Beech seed origin and experimental design

**Figure 1:**
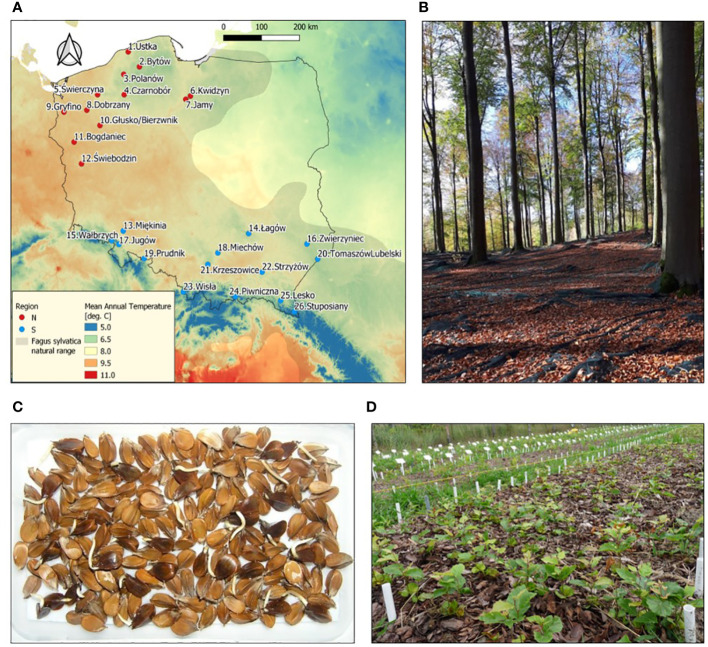
**(A)** Location of sampled *Fagus sylvatica* populations in Poland. **(B)** Seed stand in Świebodzin population. **(C)** Germination of *F*. *sylvatica* seeds after stratification at 3°C. **(D)** Common-garden nursery experiment. In **(A)** natural range of the species according to EUFORGEN (http://www.euforgen.org/species/fagus-sylvatica/) and the underlying colors show long-term (1970–2000) mean annual temperature according to WorldClim (www.worldclim.org).

The seeds for research were collected from 26 European beech stands (populations) representing different natural forests and climatic regions (6 regions and 26 mesoregions) in Poland near the north-eastern border of the natural distribution range of beech (**Supplementary Table S1**; **Figure 1**). Seeds were collected immediately after falling from the trees, dried to 9% of moisture content and stored at -3°C (Suszka et al., 1996), and their assessment was carried out according to the ISTA standards (viability using tetrazolium chloride (TTC) assay, and seed mass (1000 seeds)) (ISTA, 2019).

#### 2.1.1 Germination test

To analyze the depth of dormancy and germination traits of seeds, the seedlots (four replicates of 50 nuts per seedlot) were subjected to a stratification-germination test in a peat and soil medium (1:1) in the laboratory at 3°C. This temperature both fulfils the chilling requirements for breaking dormancy and induces seed germination in beech (Suszka, 1966; Suszka et al., 1996). Seeds were monitored once a week, and were considered germinated when the radicle protruded from the testa and pericarp. The germination was characterized by the following parameters: T_0_ - time of germination onset, germination capacity (a cumulative germination percentage at the end of the testing period), T_E_ - time at germination capacity, germination speed (a difference between T_E_ and T_0_) (El-Kassaby et al., 2008), and T_50_ - the time necessary for 50% of viable seeds to germinate (Bewley et al., 2013). T_0_ and T_50_ reflect seed dormancy depth, while germination capacity and speed reflect seed quality. Soltani et al. (2015) recommend the use of T_50_ instead of mean germination time (MGT), because it does not require a fixed germination percentage.

#### 2.1.2 Nursery common-garden test

A nursery common-garden experiment was set up in May 2021 in the experimental field of the Institute of Dendrology, Polish Academy of Sciences in Kórnik, Poland (52°14’41.0”N 17°06’10.0”E, 76 m.a.s.l.). The seeds of 24 populations (without Zwierzyniec) were sown after stratification in May 2021 in a randomized complete block design with 4 replications (blocks) of 30 seeds per population. Every second day the field was well watered with tap water.

### 2.2 Seedling growth and morphology analyses

In September 2021, 10 seedlings per population were harvested from 3 replications and measured for their height growth, root-collar diameter and above- and belowground biomass. The set of the eleven populations, representative of climatic and natural regions in Poland, was used for more detailed analysis of seedling morphological traits. Each plant was separated into leaves, stem and roots. Leaves were scanned at harvest using the Epson 7100 scanner (Epson, Nagano, Japan) and the images were then analyzed using WinSEEDLE image software (Regent Instruments Inc., Quebec, Canada). To calculate specific leaf area (SLA - the projected leaf area per unit of leaf dry biomass, m^2^ kg^−1^), leaves were dried at 105°C for 24 h and weighed.

The whole root systems were scanned in the grey scale at 300 dpi using the Epson 7100 scanner and then dried at 105°C for 24h and weighed. All analyses of root scans were done using WinRHIZO image software version 2003 (Regent Instruments Inc., Quebec, Canada). Specific root length (SRL) was determined by dividing scanned root length by its dry mass (m g^-1^). Root tissue density (RTD) was determined by dividing root dry mass by root volume (g cm^-3^).

Dry matter content of different seedling organs was determined by dividing their dry mass by fresh mass. We estimated leaf dry mass content (LDMC), stem dry mass content (SDMC), and root dry mass content (RDMC).

To estimate biomass allocation, we used the fractions of leaves (LMF; dry leaf mass/total dry mass ratio, in g g^−1^), stems (SMF; dry stem mass/total dry mass ratio, in g g^−1^), and roots (RMF; dry root mass/total dry mass ratio, in g g^−1^) in the total plant biomass following (Reich, 2002; Poorter and Sack, 2012). LMF, SMF, and RMF was calculated per each seedling.

### 2.3 Climatic data

Climatic data for maternal stands (**Supplementary Table S2**) were obtained from the ClimateEU v4.63 (Marchi et al., 2020). The data included monthly, seasonal and annual climate variables from the period between 1991 and 2020. Because we observed the differences between seed masting for the two regions of Poland (North vs. South), we also checked the hypothesis whether seed masting is associated with short-term weather conditions of the year preceding and the year current to seed crop. For that purpose we obtained monthly data of the same climatic parameters for the years 2018–2020.

### 2.4 Statistical analyses

The seed traits obtained from the stratification-germination test and seedling traits from the nursery experiment were subject to ANOVA. Normality of data distribution was checked by Shapiro-Wilk test. Data that did not meet the assumption of normality (LDMC, LMF, SMF, SRL, and RTD) were subjected to Box-Cox transformation due to skewness of distributions. Two separate ANOVAs were performed – one to test for differences among populations, followed by the Tukey’s HSD test, and the other to test for differences between the two regions of northern (N) and southern (S) Poland based on population mean values, followed by the t-test (using a significance level of P ≤ 0.05).

**Figure 2:**
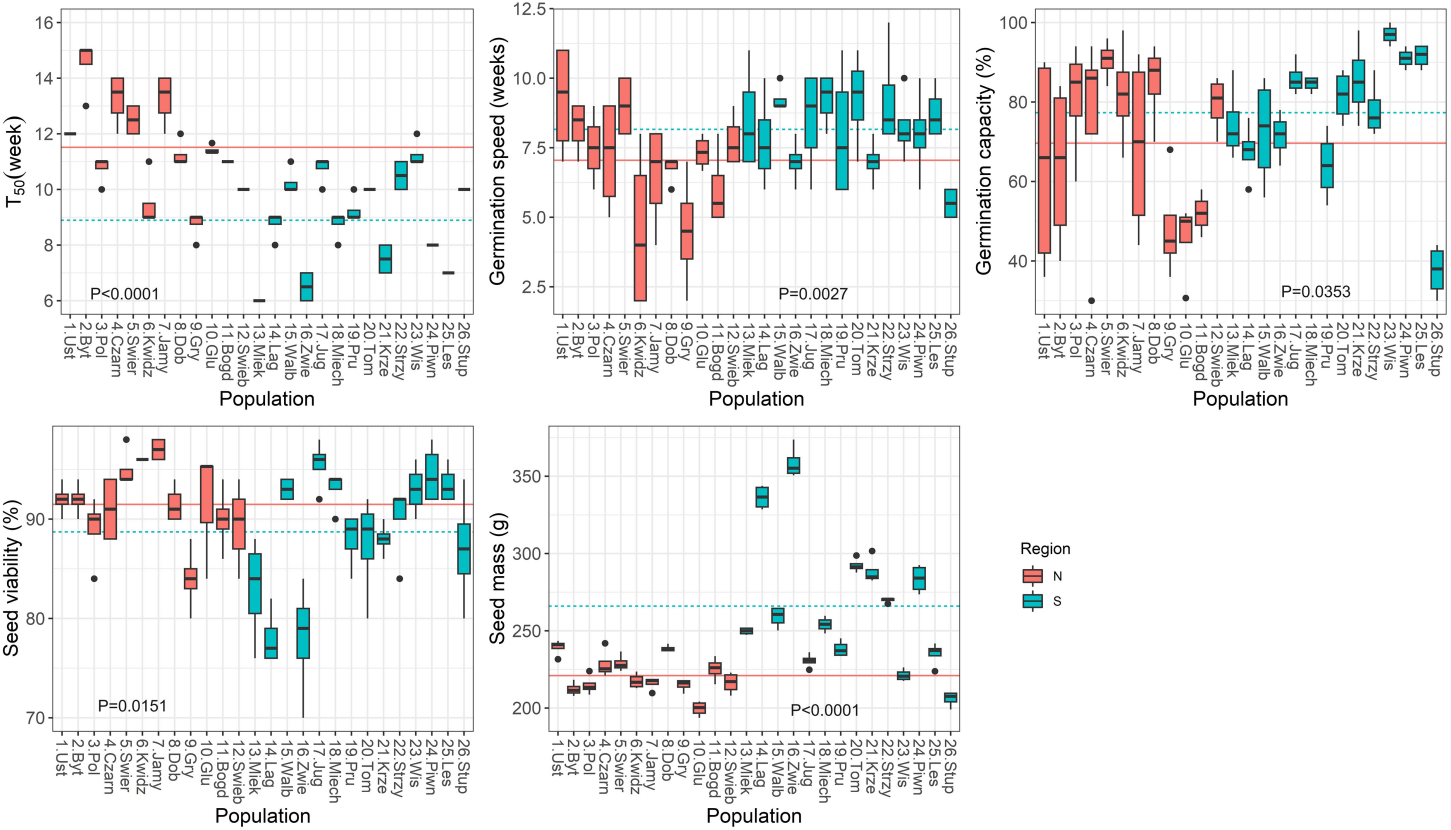
Boxplots showing variation among populations in parameters obtained from the germination test. Lines show average values for two regions of population origin. The differences between regions were significant at the P ≤ 0.05 (t-test). T_50_ (the time necessary for 50% of viable seeds to germinate).

Correlation analysis was used to test the direction and strength of relationships between climatic variables and seed germination and seedling traits, and to determine the strength of the interdependence between seed characteristics and seedlings traits. The population from Stuposiany had the lowest germination capacity (<40%, **Figure 2**) and did not emerge in the nursery, thus it was excluded from these analyses.

From among 20 annual climatic variables we removed those which had the strongest correlation (r ≥ |0.96|) with other climatic variables and the weakest correlations with germination traits. Thus, the following climatic variables were used for those analyses: MAT (mean annual temperature, °C), MWMT (mean warmest month temperature, °C), MCMT (mean coldest month temperature, °C), TD (temperature difference between MWMT and MCMT, or continentality, °C), MAP (mean annual precipitation, mm), MSP (mean summer, May to Sept., precipitation, mm), AHM (annual heat:moisture index (MAT+10)/(MAP/1000)), SHM (summer heat:moisture index ((MWMT)/(MSP/1000))). We also included the derived variables: bFFP, eFFP (beginning and end of frost-free period, respectively), PAS (precipitation as snow (mm) between August in the previous year and July in the current year), EMT (extreme minimum temperature over 30 years), E_ref_ (Hargreaves reference evaporation. For correlation analysis between germination parameters, seed and seedling traits and climatic variables the population mean value was used (N=25 or 24, depending on trait). For the correlations between seedling morphology traits and climatic variables the average values for 11 populations at three replications were used (N=33).

## 3 Results

### 3.1 Seed harvesting and weather conditions

**Figure 3:**
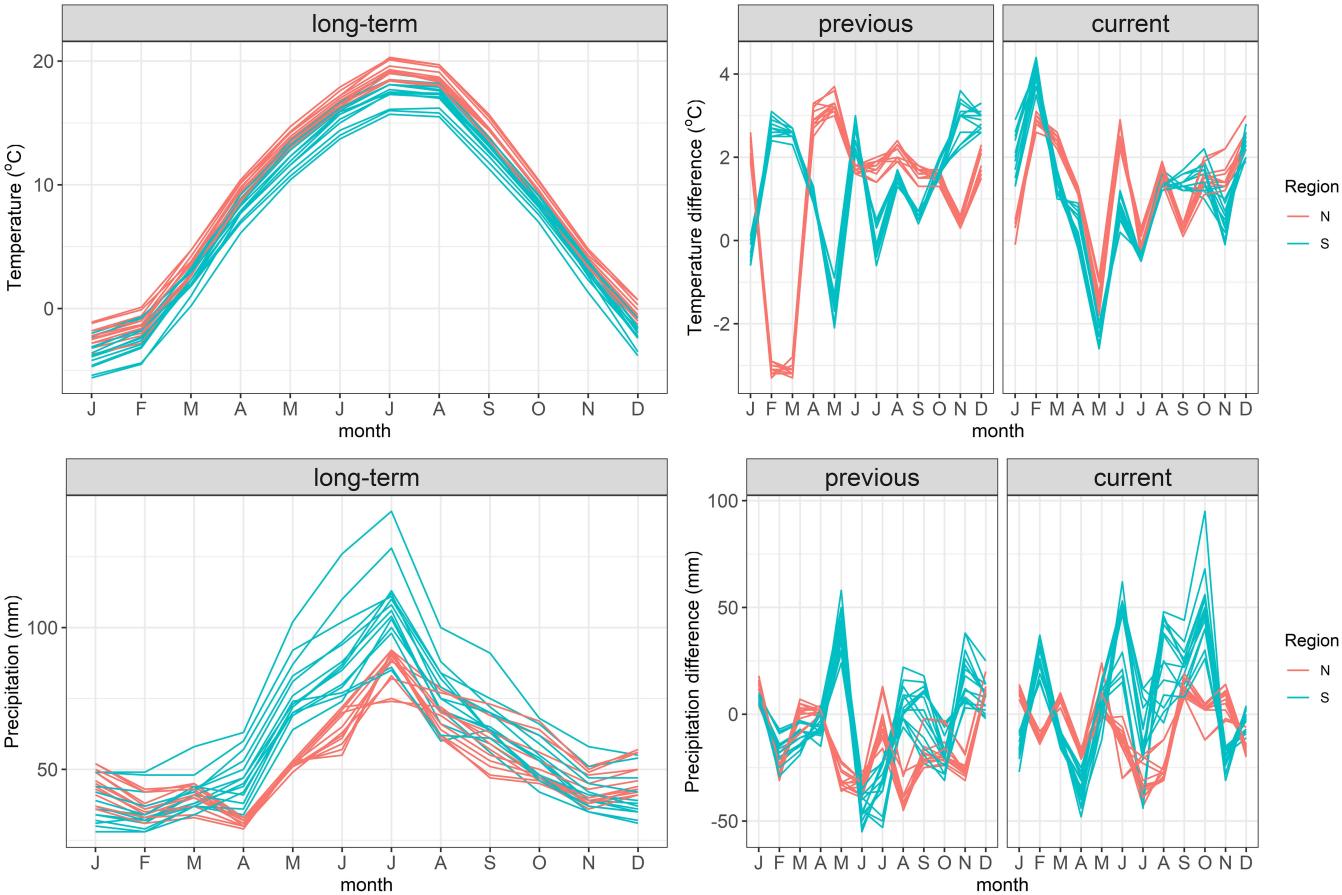
The annual time-course of the long-term (decadal 2011–2020) mean air temperatures and precipitation at the origin of beech stands sampled in the study. Data for the years previous to seed collection (one year before seed collection) and current to seed collection (year of collection) are shown as deviations from the long-term data.

The two regions differed in the year of masting. In the North a heavy mast-year occurred in 2019, without any seed crop in the South during that time. In the following year (2020) no seed was produced in the North, but a regular mast-year occurred in the South. The analysis of weather during the previous and current growing seasons revealed some common weather patterns in both regions (**Figure 3**): June of the previous-year was warmer than the long-term average, and June, July and October of the previous year were drier than average. In the current year in both regions February was warmer and May was cooler than average; April was drier, but May and September were wetter than the long-term means. Thus, the mild end of winter and cool spring, as well as the moist end of summer of the current year, but dry mid-summer of the previous year were favorable for beech seed production.

### 3.2 Germination parameters vs climatic variables at the population origin

Significant variation (P ≤ 0.0001) among populations was found for all germination parameters during the germination test (**Supplementary Table S3**; **Figure 2**). Variation was also statistically significant between the two regions of seed origin, except for the T_E_, germination capacity and seed viability. The group of northern populations started to germinate later (T_0_, by 3 weeks, **Supplementary Figure S1**), and had a longer T_50_ (by 3 weeks), but lower speed of germination (by 1 week, **Figure 2**) than the group of southern populations.

The analysis of relationships between seed germination parameters and climatic variables at the seed origin showed that germination capacity was negatively correlated with temperature and moisture availability parameters (MAT, MCMT, EMT, AHM, SHM) and positively with precipitation parameters (MAP, MSP, PAS). The strongest correlations were observed for the T_50_ and the T_0_ with E_ref_ and bFFP (negative) and eFFP (positive), but overall the strength of correlations between germination parameters and climatic variables was moderate (**Table 1**; **Supplementary Figures S2**, **S3**).

**Table 1:** Pearson correlation coefficient between germination parameters, seed and seedling traits and climatic variables at the site of population origin.

	MAT	MWMT	MCMT	TD	MAP	MSP	AHM	SHM	bFFP	eFFP	PAS	EMT	Eref	N
**seed viability**	-0.067	-0.023	0.006	-0.029	0.292	0.188	-0.244	-0.112	0.000	0.047	0.273	0.029	-0.327	25
**seed mass**	-0.347	-0.226	**-0.470**	0.223	-0.009	0.189	-0.139	-0.286	0.291	**-0.497**	0.030	**-0.516**	**0.498**	25
**T_0_**	**0.403**	**0.421**	0.376	0.042	-0.219	**-0.412**	0.318	**0.497**	**-0.543**	**0.566**	-0.140	**0.534**	**-0.626**	25
**T_E_**	0.181	0.255	0.108	0.144	0.058	-0.108	-0.011	0.172	-0.382	0.345	0.085	0.236	**-0.494**	25
**germination speed**	-0.307	-0.216	-0.382	0.164	0.412	0.435	-0.484	-0.459	0.197	-0.289	0.339	**-0.413**	0.143	25
**T_50_**	0.336	**0.401**	0.249	0.149	-0.030	-0.259	0.131	0.362	**-0.522**	**0.513**	0.022	**0.416**	**-0.622**	25
**germination** **capacity**	**-0.463**	-0.349	**-0.473**	0.123	**0.595**	**0.624**	**-0.618**	**-0.589**	0.394	**-0.484**	**0.528**	**-0.530**	0.181	25
**shoot length**	-0.323	-0.218	**-0.451**	0.227	**0.424**	**0.410**	**-0.471**	**-0.408**	0.239	-0.411	0.413	**-0.498**	0.308	24
**root length**	**-0.496**	**-0.490**	-0.398	-0.092	0.317	**0.413**	**-0.405**	**-0.468**	**0.488**	**-0.499**	0.312	**-0.495**	0.252	24
**collar diameter**	**-0.412**	-0.302	**-0.517**	0.209	**0.428**	**0.429**	**-0.509**	**-0.465**	0.304	**-0.443**	**0.440**	**-0.523**	0.249	24
**total seedling mass**	-0.335	-0.235	-0.401	0.160	0.369	0.393	**-0.406**	-0.386	0.290	**-0.415**	0.356	**-0.455**	0.335	24

Bold are significant values (P ≤ 0.05).

MAT (mean annual temperature, °C), MWMT (mean warmest month temperature, °C), MCMT (mean coldest month temperature, °C), TD (temperature difference between MWMT and MCMT, or continentality, °C), MAP (mean annual precipitation, mm), MSP (mean summer, May to Sept., precipitation, mm), AHM (annual heat:moisture index (MAT+10)/(MAP/1000)), SHM (summer heat:moisture index ((MWMT)/(MSP/1000))), bFFP, eFFP (beginning and end of frost-free period), PAS (precipitation as snow (mm) between August in the previous year and July in the current year), EMT (extreme minimum temperature over 30 years), E_ref_ (Hargreaves reference evaporation). T_0_ (time of germination onset), T_E_ (time at germination capacity) and T_50_ (the time necessary for 50% of viable seeds to germinate).

### 3.3 Seedling growth and morphology vs seed characteristics

**Figure 4:**
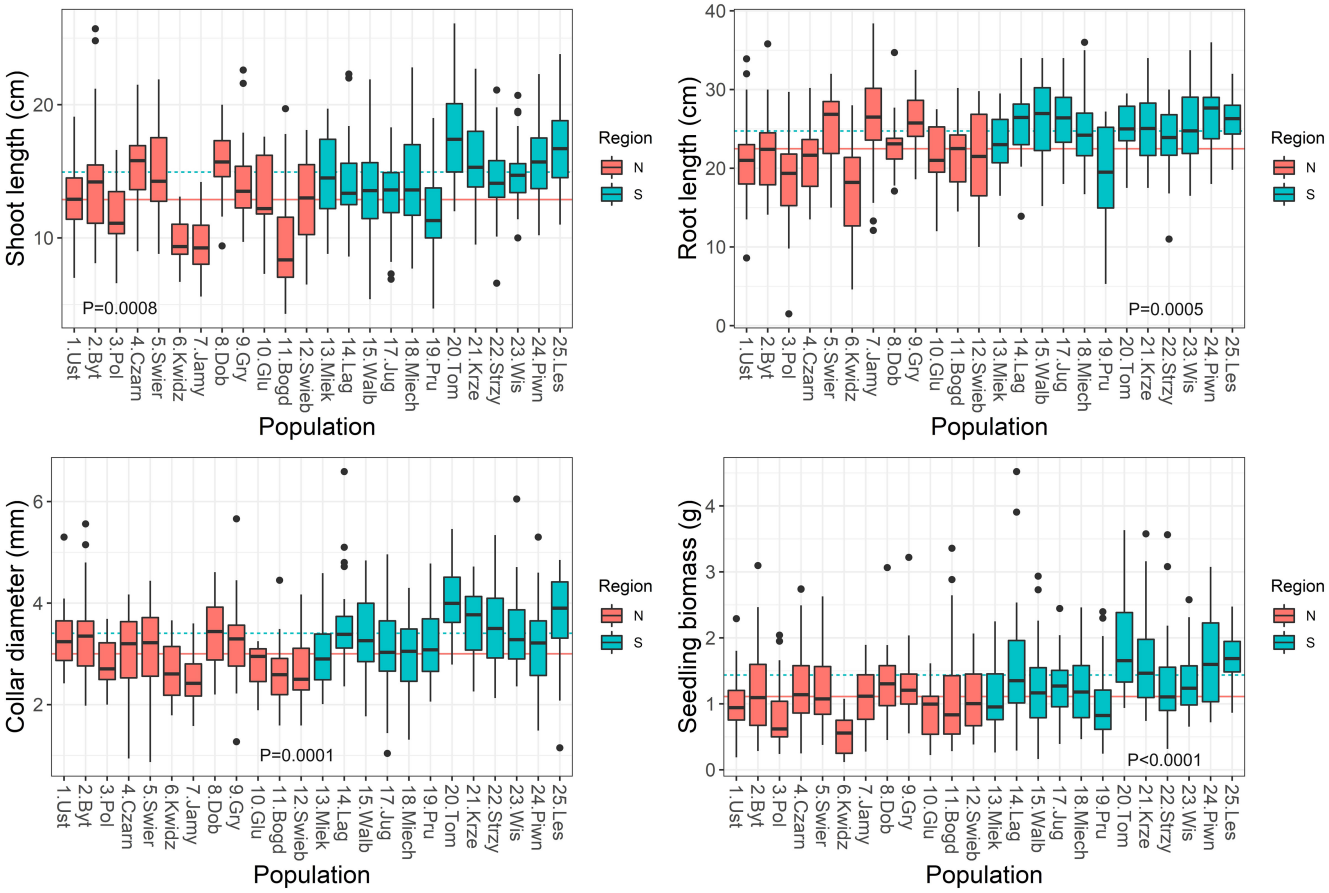
Boxplots showing variation among populations in seedling traits in the nursery. Lines show average values for two regions of population origin. The differences between regions were significant at the P ≤ 0.05 (t-test).

Significant variation was found for seedling growth and morphology in the nursery among populations and between the two regions of origin (**Supplementary Table S4**). Leaf-related traits were characterized by a low coefficient of variation: SLA (10%), LDMC (9%), LMF (20%). However, the highest variability was observed in root-related traits: SRL (44%) and RTD (31%) (**Table 2**). On average, populations from the southern region had seedlings with larger dimensions and greater final biomass than seedlings from the northern region (**Figure 4**). Seedling shoot length and diameter were negatively correlated with T_0_ and positively correlated with germination speed. Shoot length and total seedling mass were positively correlated with germination capacity, and all seedling traits except root length, were positively correlated with seed mass (r ≥ |0.42|, P ≤ 0.05) (**Supplementary Table S5**).

**Table 2:** Mean and SE (in brackets) values of seedling traits for 11 populations of *Fagus sylvatica* growing in the nursery common-garden experiment.

Population	Leaf traits			Stem traits		Root traits			
	LDMC	LMF	SLA	SDMC	SMF	RDMC	RMF	SRL	RTD
**Ustka**	0.47 (0.1) **AB**	23.76 (0.93) **AB**	170.56 (4.07) **AB**	0.47 (0.1) **ABC**	24.52 (1.22) **B**	0.39 (0.1) **A**	51.72 (1.56) **B**	6.48 (0.84) **BC**	1.32 (0.09) **A**
**Kwidzyn**	0.45 (0.1) **B**	28.03 (1.51) **A**	161.73 (4.57) **AB**	0.45 (0.3) **ABC**	28.24 (1.83) **AB**	0.34 (0.1) **ABCD**	43.73 (2.18) **C**	9.76 (0.96) **A**	0.84 (0.06) **CD**
**Dobrzany**	0.45 (0.1) **B**	21.33 (0.76) **BCD**	178.49 (2.58) **A**	0.44 (0.1) **C**	27.13 (0.91) **AB**	0.35 (0.1) **BC**	51.33 (1.27) **B**	9.01 (0.76) **AB**	1.06 (0.05) **ABC**
**Bogdaniec**	0.46 (0.1) **B**	22.84 (0.71) **ABC**	165.13 (5.99) **AB**	0.46 (0.1) BC	15.67 (0.69) **C**	0.39 (0.1) **AB**	61.49 (1.04) **A**	5.94 (0.46) **C**	1.17 (0.06) **AB**
**Świebodzin**	0.47 (0.1) **AB**	21.73 (1.52) **BCD**	164.98 (5.52) **AB**	0.47 (0.1) **ABC**	26.63 (2.84) **AB**	0.37 (0.1) **ABC**	51.65 (2.80) **BC**	7.68 (1.16) **ABC**	1.04 (0.09) **ABCD**
**Miękinia**	0.50 (0.1) **A**	21.17 (0.80) **BCD**	168.93 (3.98) **AB**	0.49 (0.1) **A**	27.33 (1.35) **AB**	0.36 (0.1) **ABC**	51.50 (1.37) **B**	9.48 (0.66) **A**	0.94 (0.05) **BCD**
**Jugów**	0.44 (0.1) **B**	20.12 (0.72) **CD**	168.31 (3.38) **AB**	0.46 (0.1) **BC**	24.30 (0.75) **B**	0.35 (0.1) **ABC**	55.58 (1.15) **B**	7.28 (0.61) **ABC**	1.01 (0.06) **ABCD**
**Miechów**	0.44 (0.1) **B**	20.09 (0.73) **CD**	174.13 (2.93) **AB**	0.47 (0.1) **AB**	24.77 (0.87) **AB**	0.36 (0.1) **ABC**	55.14 (1.11) **B**	7.71 (0.59) **ABC**	0.97 (0.04) **BCD**
**Tomaszów Lubelski**	0.46 (0.1) **B**	18.73 (0.43) **D**	171.23 (3.06) **AB**	0.46 (0.1) **BC**	29.64 (1.14) **A**	0.35 (0.1) **CD**	51.63 (1.15) **B**	9.15 (0.73) **A**	0.88 (0.05) **CD**
**Wisła**	0.46 (0.1) **B**	20.34 (0.66) **BCD**	169.56 (4.28) **AB**	0.46 (0.2) **ABC**	25.80 (1.05) **AB**	0.34 (0.1) **CD**	53.86 (1.35) **B**	9.76 (0.65) **A**	0.85 (0.06) **CD**
**Lesko**	0.46 (0.1) **B**	19.22 (0.56) **D**	161.56 (3.00) **B**	0.44 (0.1) **C**	29.98 (1.04) **A**	0.31 (0.1) **D**	50.80 (1.15) **BC**	9.83 (0.63) **A**	0.80 (0.03) **D**
***P***	*< 0.0001*	*< 0.0001*	*0.0442*	*< 0.0001*	*< 0.0001*	*< 0.0001*	*< 0.0001*	*< 0.0001*	*< 0.0001*

Values matched with the same letters do not differ statistically within column (Tukey test, P <0.05).

LDMC (leaf dry mass content), leaf mass fraction (LMF), specific leaf area (SLA), SDMC (stem dry mass content), SMF (stem mass fraction), RDMC (root dry mass content), RMF (root mass fraction), specific root length (SRL), RTD (root tissue density).

Seed viability showed a negative association with the dry matter content of leaves and stems, while it had no significant effect on the other traits including root-related parameters (**Table 3**). Later seed germination time (T_0_ and T_50_) negatively affected leaf (LDMC, but not LMF) and stem traits, but positively affected root traits (RDMC, RMF and RTD), except SRL. Faster germination (greater germination speed) was associated with lower biomass allocation to leaves (LMF), but positive correlations were found for SMF. In general, however, those correlations between seed germination parameters and seedling morphology were weak (r ≤ |0.47|; **Table 3**).

**Table 3:** Pearson correlation coefficient between seed properties and seedling morphology and biomass allocation traits growing in the nursery test (N=11).

	Leaf traits			Stem traits		Root traits			
	SLA	LDMC	LMF	SDMC	SMF	RDMC	RMF	SRL	RTD
**seed viability**	-0.06	**-0.29**	0.05	**-0.25**	-0.10	-0.07	0.06	-0.10	0.02
**germination capacity**	0.01	-0.09	**-0.26**	**-0.13**	**0.47**	**-0.37**	**-0.25**	**0.31**	**-0.34**
**T_0_**	0.11	**-0.16**	**0.22**	**-0.13**	**-0.27**	**0.23**	0.11	**-0.22**	**0.29**
**T_E_**	**0.13**	**-0.15**	0.01	-0.06	-0.10	**0.20**	0.10	**-0.21**	**0.26**
**germination speed**	0.03	0.03	**-0.32**	0.12	**0.27**	-0.08	-0.03	0.05	-0.08
**T_50_**	0.11	**-0.17**	**0.15**	**-0.13**	**-0.25**	**0.22**	**0.15**	**-0.23**	**0.27**
**seed mass**	0.10	0.01	-0.24	0.03	0.24	-0.05	-0.07	0.07	-0.10

Bold are significant values (P ≤ 0.05).

LDMC (leaf dry mass content), leaf mass fraction (LMF), specific leaf area (SLA), SDMC (stem dry mass content), SMF (stem mass fraction), RDMC (root dry mass content), RMF (root mass fraction), specific root length (SRL), RTD (root tissue density). T_0_ (time of germination onset), T_E_ (time at germination capacity) and T_50_ (the time necessary for 50% of viable seeds to germinate).

### 3.4 Seedling growth and morphological traits vs climatic variables

The relationships between seedling growth traits and climatic data at the seed origin were moderate or weak (**Table 1**). Seedling collar diameter and root length were negatively correlated with MAT, EMT, AHM and SHM. In addition, correlations between root length and MWMT, eFFP (negative) and bFFP (positive) were significant. The positive correlations were found between stem diameter and shoot length and MAP and MSP (**Table 1**; **Supplementary Figure S4**). Overall, the pattern of correlations indicates that better growth of seedlings after germination in the nursery was associated with cooler and moister conditions at the site of seed origin.

In general, the relationships between seedling morphological and allocational traits and climatic data at the seed origin were moderate or weak (**Table 4**). The LDMC, SLA, SDMC and RMF did not correlate significantly with any climatic factor (except RMF vs. E_ref_; **Table 4**). LMF had significant correlations with most of climatic variables - it increased over the temperature scale from cold to warm sites (MAT, MWMT, MCMT), but decreased with MAP, MSP and E_ref_. The SMF correlated negatively with the temperature and AHM, and positively with PAS (**Table 4**; **Supplementary Figure S5**).

**Table 4:** Correlation coefficients (r) and P values for the relationships between seedlings traits of 11 sampled beech populations in the nursery common-garden experiment and climatic variables at the origin site.

		Leaf traits			Stem traits		Root traits				
Climatic variable		LDMC	LMF	SLA	SDMC	SMF	RDMC	RMF	SRL	SRA	RTD
MAT	r	-0.055	**0.415**	0.208	0.056	**-0.440**	**0.627**	0.163	**-0.427**	**-0.549**	**0.658**
	P	0.7628	**0.0164**	0.2543	0.7583	**0.0104**	**<0.0001**	0.3659	**0.0133**	**0.0009**	**<0.0001**
MWMT	r	-0.128	**0.422**	0.159	-0.068	-0.330	**0.523**	0.059	**-0.348**	**-0.457**	**0.582**
	P	0.4776	0.0144	0.3846	0.7079	0.0611	**0.0018**	0.7463	**0.0471**	**0.0074**	**0.0004**
MCMT	r	0.099	**0.431**	0.206	0.221	**-0.447**	**0.650**	0.160	**-0.408**	**-0.521**	**0.617**
	P	0.5819	**0.0123**	0.2578	0.2175	**0.0091**	**<0.0001**	0.3740	**0.0183**	**0.0019**	**0.0001**
TD	r	-0.282	0.039	-0.039	-0.347	0.100	-0.088	-0.112	0.030	0.021	0.026
	P	0.1115	0.8298	0.8334	0.0481	0.5815	0.6243	0.5335	0.8664	0.9066	0.8838
MAP	r	-0.055	**-0.369**	-0.021	-0.049	0.254	**-0.356**	-0.020	0.314	0.341	-0.306
	P	0.7615	**0.0345**	0.9094	0.7859	0.1535	**0.0421**	0.9109	0.0748	0.0520	0.0833
MSP	r	-0.007	**-0.448**	-0.057	0.038	0.251	**-0.411**	0.028	0.329	**0.389**	**-0.422**
	P	0.9697	**0.0090**	0.7558	0.8347	0.1596	**0.0175**	0.8788	0.0616	**0.0252**	**0.0144**
AHM	r	0.035	**0.440**	0.064	0.055	**-0.396**	**0.492**	0.108	**-0.390**	**-0.451**	**0.444**
	P	0.8477	**0.0103**	0.7287	0.7627	**0.0225**	**0.0036**	0.5479	**0.0248**	**0.0084**	**0.0097**
SHM	r	-0.050	**0.496**	0.094	-0.070	-0.364	**0.508**	0.048	**-0.381**	**-0.470**	**0.534**
	P	0.7817	**0.0033**	0.6084	0.6976	0.0371	**0.0025**	0.7914	**0.0287**	**0.0057**	**0.0014**
bFFP	r	0.152	**-0.435**	-0.237	0.050	0.261	**-0.544**	0.011	0.354	**0.467**	**-0.619**
	P	0.3994	**0.0114**	0.1919	0.7814	0.1429	**0.0011**	0.9501	0.0436	**0.0061**	**0.0001**
eFFP	r	-0.053	**0.510**	0.202	0.029	**-0.373**	**0.620**	0.048	**-0.407**	**-0.548**	**0.710**
	P	0.7686	**0.0024**	0.2688	0.8710	**0.0325**	**0.0001**	0.7918	**0.0187**	**0.0010**	**<0.0001**
FFP	r	-0.106	**0.479**	0.223	-0.012	-0.320	**0.590**	0.018	**-0.386**	**-0.515**	**0.674**
	P	0.5585	**0.0048**	0.2190	0.9480	0.0692	**0.0003**	0.9225	**0.0266**	**0.0022**	**<0.0001**
PAS	r	-0.071	**-0.353**	-0.197	-0.248	**0.391**	**-0.567**	-0.154	**0.395**	**0.469**	**-0.489**
	P	0.6951	**0.0441**	0.2792	0.1646	**0.0243**	**0.0006**	0.3916	**0.0227**	**0.0059**	**0.0039**
EMT	r	0.029	**0.537**	0.200	0.112	-0.381	**0.631**	0.040	**-0.392**	**-0.510**	**0.633**
	P	0.8723	**0.0013**	0.2718	0.5361	0.0285	**<0.0001**	0.8246	**0.0241**	**0.0024**	**<0.0001**
Eref	r	0.031	**-0.507**	-0.108	0.082	-0.257	-0.046	**0.522**	-0.099	-0.038	-0.140
	P	0.8640	**0.0026**	0.5559	0.6521	0.1485	0.8005	**0.0018**	0.5843	0.8325	0.4373

Bolded are significant values (N=33).

LDMC (leaf dry mass content), leaf mass fraction (LMF), specific leaf area (SLA), SDMC (stem dry mass content), SMF (stem mass fraction), RDMC (root dry mass content), RMF (root mass fraction), specific root length (SRL), RTD (root tissue density). MAT (mean annual temperature, °C), MWMT (mean warmest month temperature, °C), MCMT (mean coldest month temperature, °C), TD (temperature difference between MWMT and MCMT, or continentality, °C), MAP (mean annual precipitation, mm), MSP (mean summer, May to Sept., precipitation, mm), AHM (annual heat:moisture index (MAT+10)/(MAP/1000)), SHM (summer heat:moisture index ((MWMT)/(MSP/1000))), bFFP, eFFP (beginning and end of frost-free period), PAS (precipitation as snow (mm) between August in the previous year and July in the current year), EMT (extreme minimum temperature over 30 years), E_ref_ (Hargreaves reference evaporation).

Considering root traits, seedlings allocated a similar fraction of biomass belowground (RMF) regardless of climatic conditions at the site of their origin (**Tables 2**, **4**). However, the other root traits showed significant correlations with climate. Temperature at the sites of origin exhibited strong positive effects on RDMC and RTD (**Supplementary Figure S5**) that increased from the coldest to warmest sites, whereas a negative effect was reflected by a decline of SRA and SRL at the warmer sites (**Table 4**; **Supplementary Figure S5**). Root traits were only weakly affected by precipitation-related parameters. However, populations originating from sites with lower water availability (higher AHM and SHM) produced seedlings with shorter and tougher roots than those from sites with more favorable climatic moisture (**Table 2**, **4**; **Supplementary Figure S5**).

## 4 Discussion

The present study showed that beech masting differed between the regions of Poland. Similar differences have been observed for beech since 1945 (Białobok, 1990). We analyzed the correlation between masting and weather in the previous year (when the floral buds were formed) and the current year (when flowering and seed formation occurred) and revealed some common patterns for both regions in temperature and precipitation. The cool spring and the moist summer of the current year, but dry summer of the previous year were favorable for beech seed production. Observed differences in beech masting between regions of Poland may be the results of climate variability. Climate conditions and their disturbances influence the masting of trees (Hacket-Pain and Bogdziewicz, 2021). Bogdziewicz et al. (2020a) implied that climate change is the factor which increases interannual variability of seed production in beech. Analysis of beech masting in England showed that climate warming increased seed production, however, it also decreased the interannual variability and synchrony of seed production which suggests a threat to natural beech regeneration (Bogdziewicz et al., 2020b). As global temperatures are expected to increase during this century, trees that fine-tune their reproductive schedules based on temperature cues may suffer regeneration failures (Bogdziewicz et al., 2021). Our observation of beech masting in Poland during the last years (unpublished data) showed some disturbances in masting, which may imply the influence of climate warming. Especially lower summer precipitation could be the cause of the changes. Northern beech populations can suffer much more because of less water availability. Present results indicate that spatial synchronicity of massive seed production in beech may also vary regionally due to spatio-temporal variation of environmental cues, but whether this variation shows any trend with changing climate remains to be examined in more detail.

The present study described how adaptation to climatic conditions of populations’ habitats influence seed dormancy and germination traits of European beech. We showed that seed traits differed among populations and regions of seed origins. The northern populations had deeper seed dormancy and required longer stratification for germination, but had greater seed viability and germination speed, and lower germination capacity and seed mass than the group of southern populations. The link between tree seed germination traits and provenance was also observed for beech in Greece (Varsamis et al., 2020) and across Europe (Muffler et al., 2021), for *Quercus ilex* in the Iberian Peninsula and Morocco (García-Nogales et al., 2016), *Acer pseudoplatanus* in Europe (Daws et al., 2006), *Pinus brutia* in Greece (Skordilis and Thanos, 1995), *Alnus* species in Europe (Gomes Marques et al., 2022) and *Betula pendula* in Europe (Midmore et al., 2015; Solé-Medina et al., 2020). Varsamis et al. (2020) observed that beech seeds from warmer and wetter provenances started to germinate earlier during stratification, suggesting a nondeep dormancy level. Higher mean annual temperature could shape the course of germination by rendering the development of deep seed dormancy unnecessary. They suggested that water availability can also be a factor influencing depth of seed dormancy (Varsamis et al., 2020). The link between germination and latitude was shown for the evergreen oak (*Quercus ilex*) where the southern populations germinated earlier, and this trait was related mainly to climatic conditions (García-Nogales et al., 2016). Our results also show that seed dormancy in beech is influenced by the climate of seed origin site and latitude – surprisingly however, less by the mean annual temperature or precipitation, but more so by the beginning and end of frost-free period (bFFP, eFFP) and reference evaporation (E_ref_).

Seed dormancy and germination traits are related to plant fitness through their effects on timing and speed of seedling emergence. Our study showed that climate affects the seed germination traits of beech populations. The strongest negative influences on dormancy depth were found for frost and evaporation. The germination capacity was positively correlated with precipitation, and negatively with temperature and moisture deficit at the seed origin site. These results indicate that dormancy and germination traits of seeds driven by a historical environment are limited by water deficit and temperature conditions, where frost decreases dormancy depth, whereas high temperature and low moisture availability decrease germination capacity. Zhang et al. (2022) indicated that precipitation has significant negative effects whereas temperature seasonality has significant positive effects on the proportion of dormancy of woody species. In beech this was partly proven regarding the influences of evaporation and seasonality. Muffler et al. (2021) also observed that beech populations originating from the northern edge of distribution with dry and cold winters had worse germination compared to populations from regions with wetter winters that germinated to a greater extent. We conclude that germination of beech seeds depends on the climatic conditions of the provenance, associated with the seasonal variation of temperature and rainfall. The patterns of variation found in these parameters indicate adaptation of populations to the climate of seed origin site.

In the present study, we found evidence of local adaptation of seedling growth. Significant variation was found both among populations and between the two regions of seed origin. Populations from the southern region produced seedlings of greater final biomass than the northern ones. Root and shoot growth were negatively correlated with temperature and water deficit, but positively with precipitation. This indicates that better growth of seedlings was associated with cooler and moister conditions. Analysis of beech fitness in Spain also showed segregation among populations according to local climate (Robson et al., 2013). It was found that local adaptation promotes frost avoidance rather than drought prevention. The experiment of Kreyling et al. (2012) showed that late frost is dangerous for beech leaf development and seedling growth. Northern beech populations are better adapted to frost in comparison to South-European populations (Kreyling et al., 2012). Müller et al. (2020) found a low level of beech population differentiation regarding survival and height increment, but high phenotypic plasticity. Survival showed a positive correlation with temperature variables and a less pronounced and negative correlation with precipitation-related variables. Sensitivity of beech seed germination and fitness to the water supply can be explained by the suborthodox (intermediate) character of these seeds (Michalak et al., 2023). They are sensitive to long term storage in a desiccated state. Summarizing, beech seed germination and seedling development may be exposed to threatening conditions of water deficit and increased temperatures.

Analysis of correlation between seed and seedling traits indicates that fitness of beech correlates with shallow seed dormancy, higher germination capacity and heavier seed mass. The positive relationship between seed mass and plant establishment was observed for annual plants as well as woody plants, e.g. in *A. glutinosa* and *A. lusitanica* (Saatkamp et al., 2019; Gomes Marques et al., 2022). Greater seed mass positively influences germination and plant establishment due to the greater amount of reserves accumulated in seeds (Fenner and Thompson, 2005). Seed biomass also positively affects tree recruitment processes and its relevance increases with environmental harshness (Badano and Sánchez-Montes de Oca, 2022).

Analysis of morphological seedling traits showed that they differed among provenances with the highest variability observed in root-related traits. Although biomass allocation to roots was not associated with climatic conditions, the temperature of the origin sites exhibited strong positive effects on root mass and density, whereas negative effects were reflected by a shorter root length in populations from warmer sites. Production of longer and finer roots was associated with higher moisture availability at the sites of seed origin. These root traits may allow for effective water absorption from the soil, but may also reduce the ability to withstand drought (Zadworny et al., 2021). On the other hand, the root length as well as stem mass decreased, and leaf mass increased from cold to warm and dry sites. This indicates that even those shorter and tougher roots were effective in providing enough water to the aboveground parts. The study by Stojnić et al. (2015) showed that the population differences in beech leaf traits have a genetic basis. The associations between phenotype and environment indicated adaptive variation in beech growth and phenology associated with temperature and water supply (Frank et al., 2017). Solé-Medina et al. (2022) showed that phenotypic plasticity was associated with provenance climate across the range of a Mediterranean oak (*Quercus faginea*). Populations from drier and colder climates had lower growth rates, more favorable photosynthetic parameters and lower phenotypic plasticity when compared to those from milder conditions. Thus, contrasting rainfall and temperature conditions exert control over the adaptive intraspecific evolution of multivariate phenotypes and their plasticity (Solé-Medina et al., 2022). Ramírez-Valiente et al. (2023) demonstrated the climate-associated divergent selection among populations in two Mediterranean oaks, *Quercus faginea* and *Q. lusitanica*, on leaf morphology, physiology and growth rate. These observations are similar to those presented here regarding beech morphology diversity associated with population climate, however, high temperature and low water availability rather than cold are factors inhibiting growth. A study of silver birch (*Betula pendula*) indicated that populations differed in seedling traits and drier conditions limited birch regeneration capacity (Solé-Medina et al., 2020).

Our results imply that increasing temperatures and decreasing precipitation may be factors limiting beech regeneration in the future. Although individual climatic variables describing the origin sites in our study explained only a moderate part of the variation among examined beech populations in seed germination and seedling traits, there appears to be a clear pattern that populations originating from warmer and drier sites (mostly located in the northern region), when compared with those from the opposite end of climatic gradient, germinated later and with a lower success, and produced smaller seedlings with shorter and tougher roots. This trend is worrisome for several reasons. Firstly, it indicates that those populations require a longer stratification period for seed dormancy breakage, which may be disturbed by additional climatic warming, especially in winter. A longer dormancy breaking process may also deplete seed reserves, leading to further problems with germination and seedling development (Reed et al., 2022). Particularly concerning is that later-emerging seedlings may be subject to drought, especially since their root morphological traits may be less favorable for water absorption from the soil than in populations from the southern group. On the other hand, the thinner stems and tougher roots of those seedlings may contribute to their higher resistance to embolism (Domec et al., 2010).

The effects of climate change are generally expected to reduce tree growth and survival, predispose forests to disturbance by wildfire, insects and disease, and ultimately change forest structure and composition at the landscape scale (Chmura et al., 2011). Substantial warming decreases winter chilling, resulting in delayed bud burst, and adversely affecting flowering and seed formation. Thereupon seed quality and germination may be disturbed. Additionally, seeds which have deeper dormancy will require a longer cold period to germinate. The extent of warming effects will depend on the magnitude of climate change, the abilities of individual trees to acclimate, and for tree populations to adapt *in situ*, or to migrate to suitable habitats (Chmura et al., 2011). These coping mechanisms may be insufficient to maintain optimal fitness of tree populations in a rapidly changing climate. In the light of these insights and the results of our study, warming can disturb the correct progression of seed germination and in consequence beech regeneration. Water deficit and ground frost, especially in warming conditions, maybe the limiting factors for beech regeneration. Plasticity of beech populations might not be sufficient to ensure its regeneration in the future due to the extreme heat and drought events at the rear (southern) edge (Muffler et al., 2021). At the cold distribution margin, the high plasticity in the early life-history traits may allow for increasing germination success with increasing temperatures; however, warming may negatively affect seedling establishment and survival, and thus may facilitate natural regeneration in the future only to a limited extent (Muffler et al., 2021). Because of the differentiation in growth and phenological responses of beech, Vander Mijnsbrugge et al. (2021) suggested that caution should be taken when translocating provenances in anticipation of the predicted climate warming, what we can also imply grounded on the local habitat-based associations of seed and seedling properties.

## 5 Conclusions

The results of our study indicated the existence of differentiation in adaptation in seed and seedling ecology among provenances of *Fagus sylvatica* in Poland, at the northeastern range of the species distribution. This adaptation strategy is related to environmental conditions at the provenance level. The temperature and precipitation variables affecting climatic water availability shaped to some degree the seed dormancy and germination pattern among examined beech populations, but less so the variation in seedling growth and morphology. Populations originating from warmer and drier sites in our study (northern populations) required a longer period of seed stratification, germinated later and with a lower success, and produced smaller seedlings, that together would expose them to the risks associated with projected climate change. On the other hand, their root morphology may indicate adaptation to lower water availability. Understanding how beech populations adjust to new environments is critically important given the major role of northern populations in broadening their distribution range. Varying requirements for seed germination, the differences in the success of seedling emergence and seedling growth and morphology will contribute to variation in the regeneration success within the species. Studies are needed, however, to elucidate the concerted effects of variation in those traits on the long-term persistence of beech populations and their adaptation ability under changing climatic conditions.

## Data availability statement

The raw data supporting the conclusions of this article will be made available by the authors, without undue reservation.

## Author contributions

TP: Conceptualization, Data curation, Formal analysis, Funding acquisition, Methodology, Project administration, Resources, Supervision, Validation, Writing – original draft, Writing – review & editing, Visualization. JS: Conceptualization, Data curation, Investigation, Methodology, Resources, Writing – review & editing. JM: Data curation, Formal analysis, Methodology, Software, Validation, Visualization, Writing – review & editing. MZ: Data curation, Formal analysis, Methodology, Validation, Visualization, Writing – review & editing. SA: Investigation, Writing – review & editing. BK: Data curation, Writing – review & editing. PC: Investigation, Writing – review & editing. AJ: Writing – review & editing, Conceptualization. DC: Data curation, Formal analysis, Methodology, Software, Validation, Visualization, Writing – review & editing.
