# Supplementary material for: Climate legacy in seed and seedling traits of European beech populations
Source: Front Plant Sci. 2024 Jun 7;15:1355328. doi: 10.3389/fpls.2024.1355328 (PMC11190307; doi:10.3389/fpls.2024.1355328)
Supplement: Supplementary file 1 [file DataSheet_1.docx]

Supplementary Material

# Supplementary Figures and Tables

## Supplementary Figures


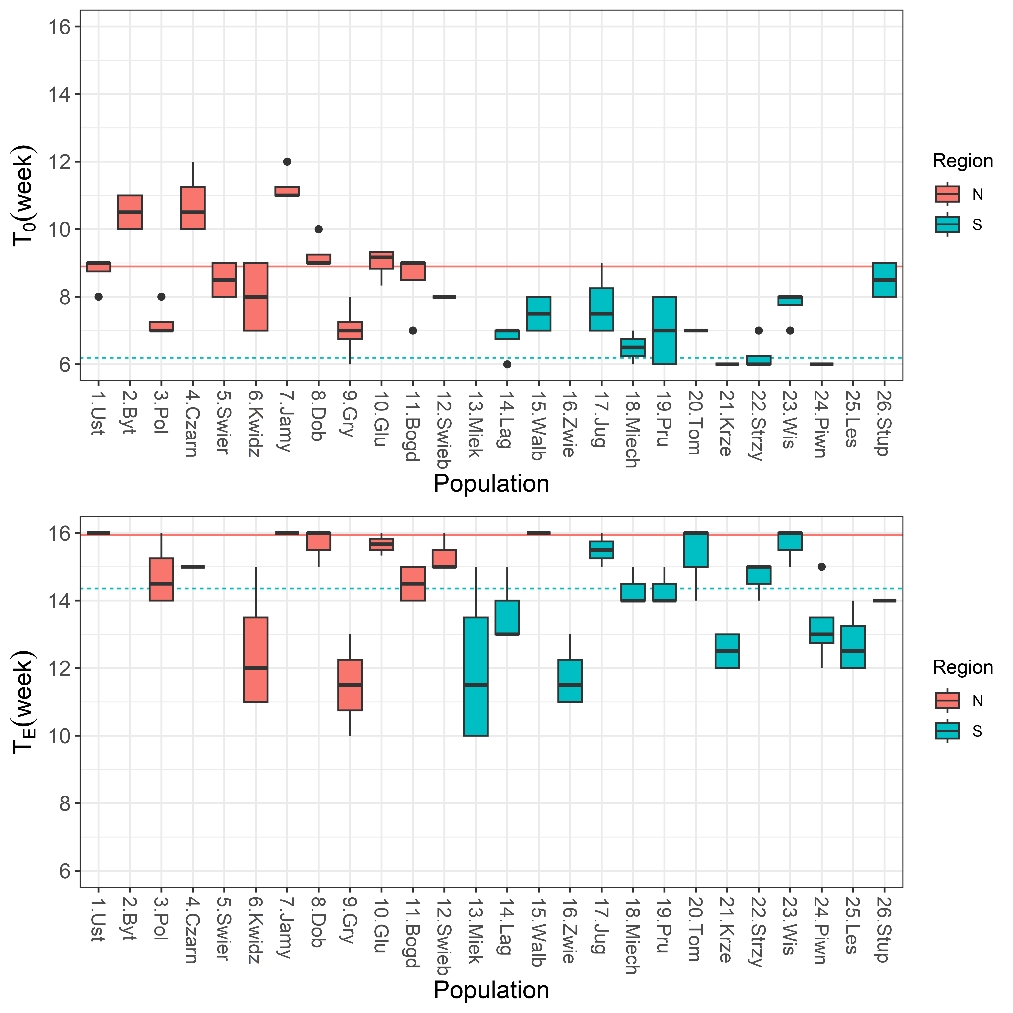


P=0.0005

P<0.0001

**Supplementary Figure S1.** Boxplots showing variation among populations in time of beginning of germination (T_0_) and end of germination (T_E_). Lines show average values for two regions of population origin. The differences between regions were significant at the P ≤ 0.05 (t-test).


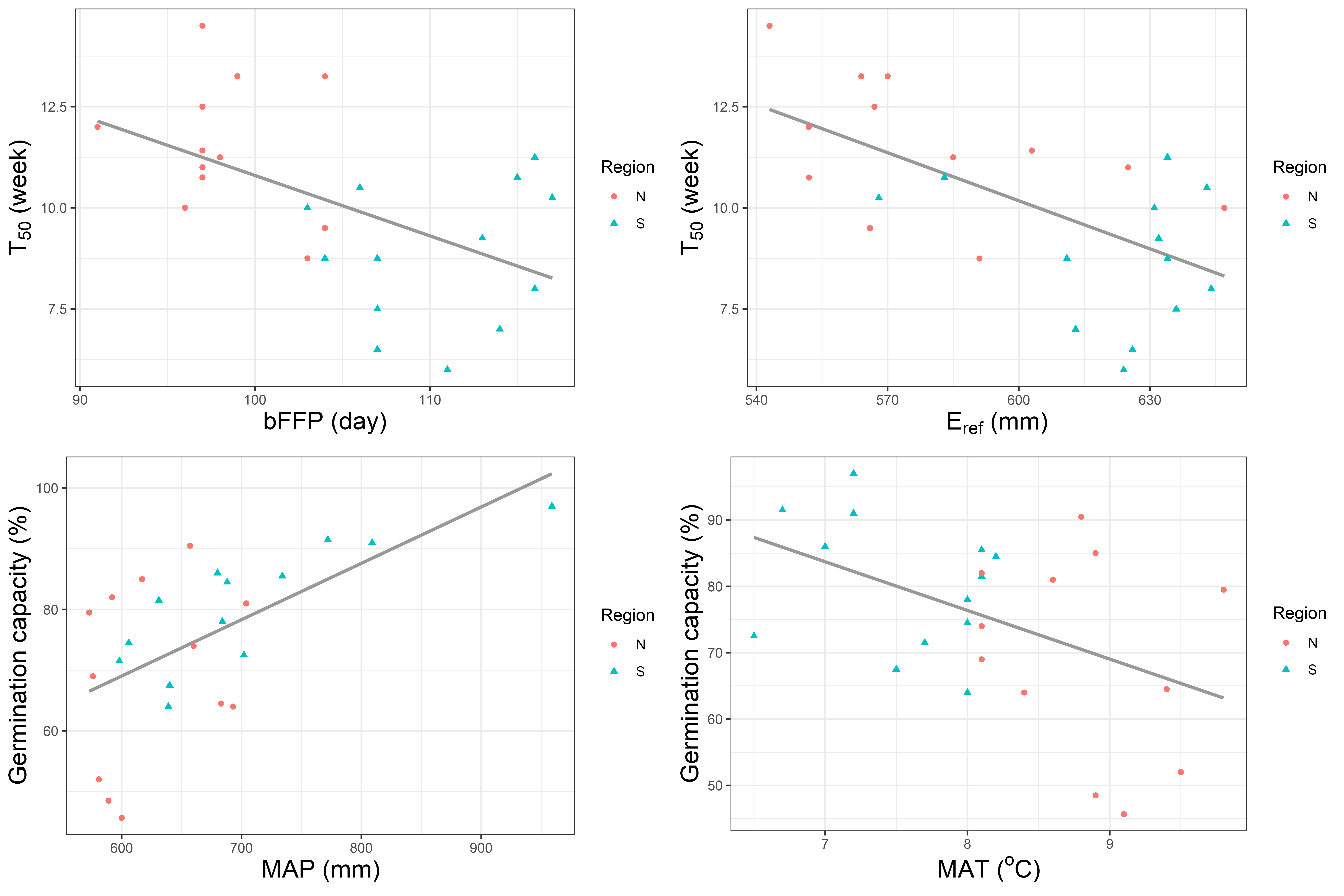


r = 0.595

p = 0.002

r = -0.463

p = 0.020

r = -0.622

p = 0.001

r = -0,522

p = 0.0074

**Supplementary Figure S2.** Analysis of correlation between beech seed germination traits and climatic conditions. bFFP (beginning of frost-free period), MAP (mean annual precipitation, mm), Eref (Hargreaves reference evaporation), MAT (mean annual temperature, °C).


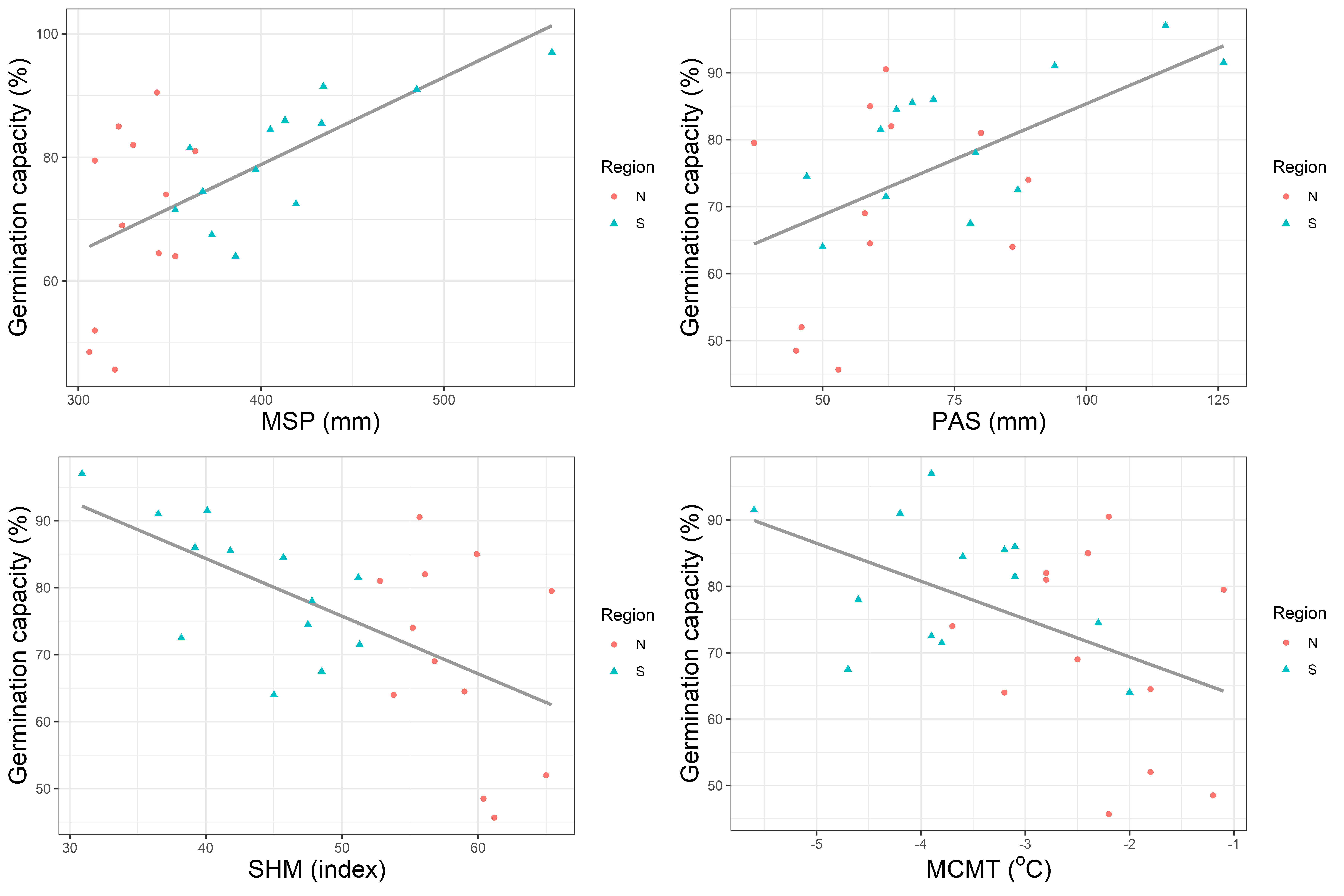


r = -0.473

p = 0.017

r = -0.589

p = 0.002

r = 0.528

p = 0.007

r = 0.624

p = 0.001

**Supplementary Figure S3.** Analysis of correlation between beech seed germination traits and climatic conditions. MSP (mean summer, May to Sept., precipitation, mm), SHM (summer heat:moisture index ((MWMT)/(MSP/1000))), PAS (precipitation as snow (mm) between August in the previous year and July in the current year), MCMT (mean coldest month temperature, °C).


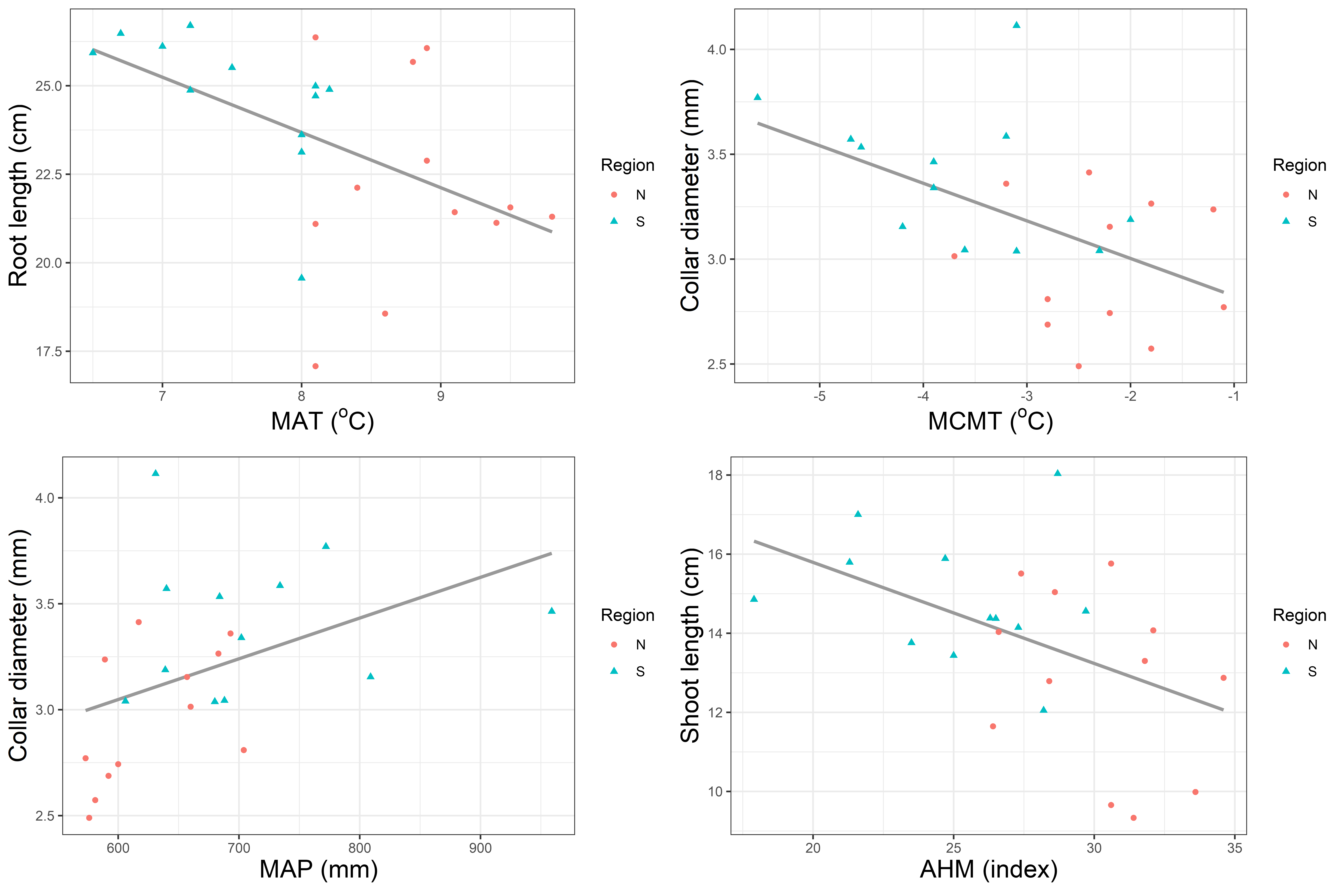


r = -0.517

p = 0.0097

r = -0.496

p = 0.0136

r = -0.471

p = 0.0202

r = 0.4278

p = 0.037

**Supplementary Figure S4.** Analysis of correlation between beech seedling growth and climatic conditions. MAT (mean annual temperature, °C), MAP (mean annual precipitation, mm), MCMT (mean coldest month temperature, °C), AHM (annual heat:moisture index (MAT+10)/(MAP/1000)).

**
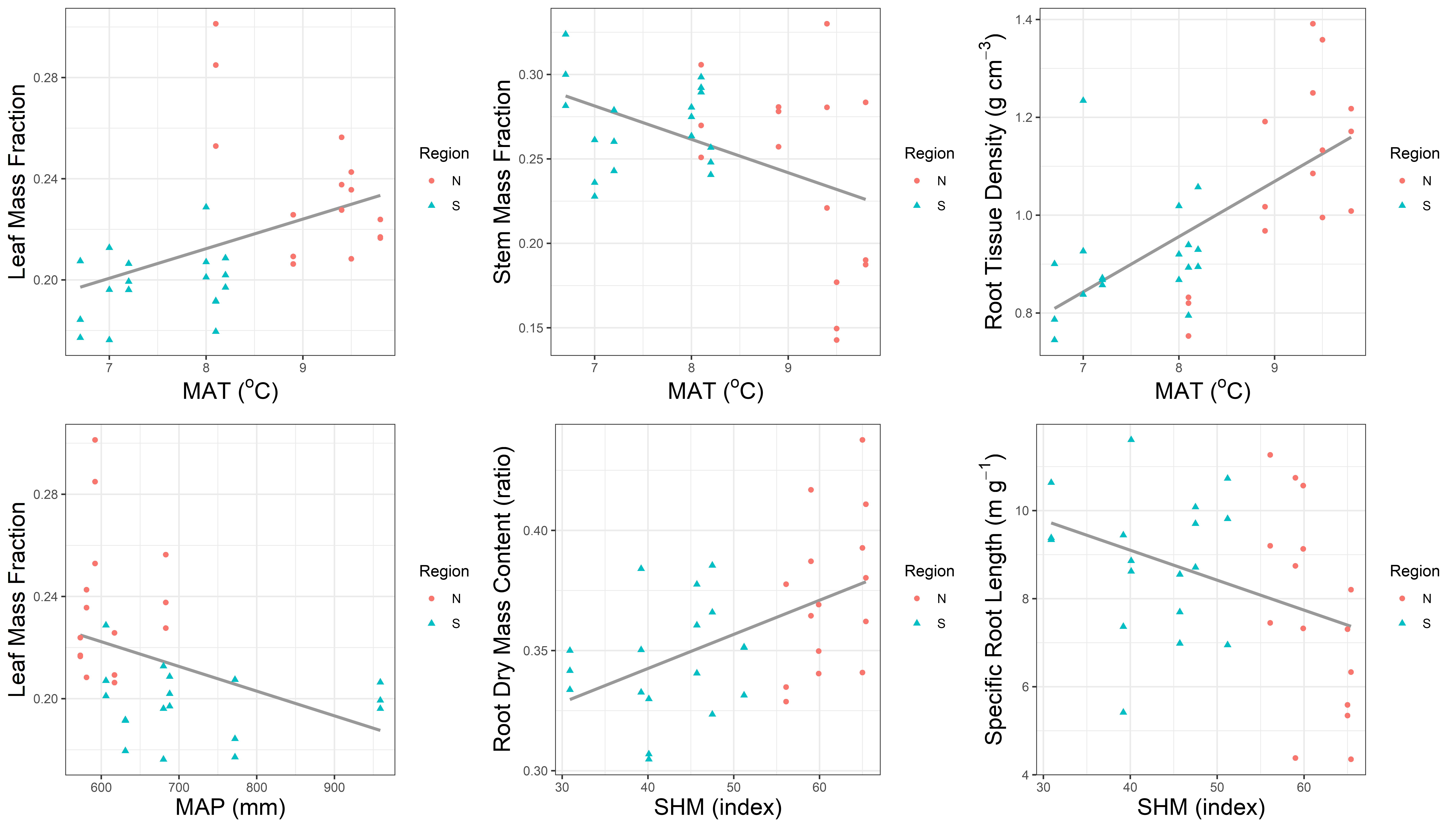
**

r = -0.440

p = 0.0104

r = 0.658

p < 0.0001

r = 0.508

p = 0.0025

r = -0.369

p = 0.0345

r = -0.381

p = 0.0287

r = 0.415

p = 0.0164

**Supplementary Figure S5.** Analysis of correlation between the biomass fraction of particular organs (leaf mass fraction, stem mass fraction), root traits characteristics (specific root length and root tissue density) and climatic variables. MAT (mean annual temperature, °C), MAP (mean annual precipitation, mm), SHM (summer heat:moisture index ((MWMT)/(MSP/1000))).

## Supplementary Tables

**Supplementary Table S1.** Population name, latitude, longitude, altitude, collection year, mean annual temperature (MAT), and mean annual precipitation (MAP) of the studied *Fagus sylvatica* populations. Populations were ordered according to descending latitude of the origin.

| **No.** | **Population name** | **Short name** | **Latitude N** | **Longitude**  **E** | **Elevation**  **(m a.s.l)** | **Collection year** | **Region** | **MAT**  **(°C)** | **MAP**  **(mm)** |
| --- | --- | --- | --- | --- | --- | --- | --- | --- | --- |
| 1 | Ustka^a^ | 1.Ust | 54.53 | 16.91 | 38.67 | 2019 | N | 9.4 | 683 |
| 2 | Bytów | 2.Byt | 54.23 | 17.3 | 113.43 | 2019 | N | 8.4 | 693 |
| 3 | Polanów | 3.Pol | 54.08 | 16.75 | 127.42 | 2019 | N | 8.6 | 704 |
| 4 | Czarnobór | 4.Czarn | 53.68 | 16.77 | 170.95 | 2019 | N | 8.1 | 660 |
| 5 | Świerczyna | 5.Swier | 53.68 | 15.83 | 112.99 | 2019 | N | 8.8 | 657 |
| 6 | Kwidzyn^a^ | 6.Kwidz | 53.66 | 19.09 | 120.39 | 2019 | N | 8.1 | 592 |
| 7 | Jamy | 7.Jamy | 53.59 | 18.93 | 108.53 | 2019 | N | 8.1 | 576 |
| 8 | Dobrzany^a^ | 8.Dob | 53.37 | 15.45 | 122.51 | 2019 | N | 8.9 | 617 |
| 9 | Gryfino | 9.Gry | 53.33 | 14.65 | 159.42 | 2019 | N | 8.9 | 589 |
| 10 | Głusko/Bierzwnik | 10.Glu | 53.06 | 15.92 | 93.45 | 2019 | N | 9.1 | 600 |
| 11 | Bogdaniec^a^ | 11.Bogd | 52.73 | 15.01 | 127.82 | 2019 | N | 9.5 | 581 |
| 12 | Świebodzin^a^ | 12.Swieb | 52.28 | 15.27 | 119.57 | 2019 | N | 9.8 | 573 |
| 13 | Miękinia^a^ | 13.Miek | 50.88 | 16.73 | 358.32 | 2020 | S | 8 | 606 |
| 14 | Łagów | 14.Lag | 50.83 | 21.13 | 388 | 2020 | S | 7.5 | 640 |
| 15 | Wałbrzych | 15.Walb | 50.68 | 16.33 | 651.79 | 2020 | S | 6.5 | 702 |
| 16 | Zwierzyniec | 16.Zwie | 50.61 | 23.17 | 310.55 | 2020 | S | 7.7 | 598 |
| 17 | Jugów^a^ | 17.Jug | 50.6 | 16.58 | 577.34 | 2020 | S | 7 | 680 |
| 18 | Miechów^a^ | 18.Miech | 50.42 | 20.05 | 349.17 | 2020 | S | 8.2 | 688 |
| 19 | Prudnik | 19.Pru | 50.29 | 17.46 | 423.84 | 2020 | S | 8 | 639 |
| 20 | Tomaszów Lubelski^a^ | 20.Tom | 50.28 | 23.55 | 286.79 | 2020 | S | 8.1 | 631 |
| 21 | Krzeszowice | 21.Krze | 50.17 | 19.7 | 432.73 | 2020 | S | 8.1 | 734 |
| 22 | Strzyżów | 22.Strzy | 50 | 21.6 | 331.9 | 2020 | S | 8 | 684 |
| 23 | Wisła^a^ | 23.Wis | 49.57 | 18.84 | 668.09 | 2020 | S | 7.2 | 959 |
| 24 | Piwniczna | 24.Piwn | 49.47 | 20.67 | 595.95 | 2020 | S | 7.2 | 809 |
| 25 | Lesko^a^ | 25.Les | 49.38 | 22.25 | 515.51 | 2020 | S | 6.7 | 772 |
| 26 | Stuposiany | 26.Stup | 49.14 | 22.74 | 850.37 | 2020 | S | 5.6 | 839 |

^a^ Populations assigned to a detailed morphological analysis in the common garden experiment.

**Supplementary Table S2.** The list of climatic variables used in the analysis. The data were derived from ClimateEU ([Marchi et al., 2020](#_ENREF_2)) for a period 1991-2020 for population origins.

| **Abbreviation** | **Explanation** |
| --- | --- |
| MAT | mean annual temperature (°C), |
| MWMT | mean warmest month temperature (°C), |
| MCMT | mean coldest month temperature (°C), |
| TD | temperature difference between MWMT and MCMT, or continentality (°C), |
| MAP | mean annual precipitation (mm), |
| MSP | mean summer (May to Sept.) precipitation (mm), |
| AHM | annual heat : moisture index (MAT+10)/(MAP/1000)) |
| SHM | summer heat : moisture index ((MWMT)/(MSP/1000)) |
| DD<0 | degree-days below 0°C, chilling degree-days |
| DD>5 | degree-days above 5°C, growing degree-days |
| DD<18 | degree-days below 18°C, cooling degree-days |
| DD>18 | degree-days above 18°C, heating degree-days |
| NFFD | the number of frost-free days |
| bFFP | the Julian date on which FFP begins |
| eFFP | the Julian date on which FFP ends |
| FFP | frost-free period |
| PAS | precipitation as snow (mm) between August in previous year and July in current year |
| EMNT | extreme minimum temperature over 30 years (°C) |
| Eref | Hargreaves reference evaporation (mm) |
| CMD | Hargreaves climatic moisture deficit (mm) |

**Supplementary Table S3.** ANOVA table for traits from the stratification-germination test for differences among populations and between the two regions of origin.

|  | **Population variation** | | | | | **Regional variation** | | | | |
| --- | --- | --- | --- | --- | --- | --- | --- | --- | --- | --- |
| **Trait** | **Source of variation** | **d.f.** | **Sum of Squares** | **F Ratio** | **Prob. > F** | **Source of variation** | **d.f.** | **Sum of Squares** | **F Ratio** | **Prob. > F** |
| **T_0_** | population | 25 | 382.6539 | 31.3409 | <0.0001 | region | 1 | 188.335 | 80.734 | <0.0001 |
|  | replication | 3 | 1.03846 | 0.7088 | 0.5498 | replication | 3 | 1.038 | 0.148 | 0.9305 |
|  | error | 75 | 36.62821 |  |  | error | 99 | 230.947 |  |  |
| **T_E_** | population | 25 | 437.7735 | 10.5814 | <0.0001 | region | 1 | 65.120 | 12.978 | 0.0005 |
|  | replication | 3 | 12.38462 | 2.4946 | 0.0664 | replication | 3 | 12.385 | 0.823 | 0.4844 |
|  | error | 75 | 124.1154 |  |  | error | 99 | 496.769 |  |  |
| **germination** | population | 25 | 194.8504 | 3.403 | <0.0001 | region | 1 | 31.966 | 9.456 | 0.0027 |
| **speed** | replication | 3 | 19.33761 | 2.8144 | 0.0449 | replication | 3 | 19.338 | 1.907 | 0.1334 |
|  | error | 75 | 171.7735 |  |  | error | 99 | 334.658 |  |  |
| **T_50_** | population | 25 | 447.6838 | 61.4777 | <0.0001 | region | 1 | 177.558 | 60.205 | <0.0001 |
|  | replication | 3 | 0.48718 | 0.5575 | 0.6447 | replication | 3 | 0.487 | 0.055 | 0.9829 |
|  | error | 75 | 21.84615 |  |  | error | 99 | 291.972 |  |  |
| **germination** | population | 25 | 23014.88 | 5.9223 | <0.0001 | region | 1 | 1525.477 | 4.556 | 0.0353 |
| **capacity** | replication | 3 | 1352.047 | 2.8993 | 0.0405 | replication | 3 | 1352.047 | 1.346 | 0.2639 |
|  | error | 75 | 11658.51 |  |  | error | 99 | 33147.908 |  |  |
| **seed mass** | population | 25 | 154396.4 | 167.4427 | <0.0001 | region | 1 | 52234.805 | 49.284 | <0.0001 |
|  | replication | 3 | 176.3782 | 1.594 | 0.1979 | replication | 3 | 176.378 | 0.056 | 0.9827 |
|  | error | 75 | 2766.256 |  |  | error | 99 | 104927.884 |  |  |
| **seed viability** | population | 25 | 2412.49 | 8.247 | <0.0001 | region | 1 | 195.139 | 6.122 | 0.0151 |
|  | replication | 3 | 40.30866 | 1.1483 | 0.3356 | replication | 3 | 37.204 | 0.389 | 0.7611 |
|  | error | 72 | 842.4847 |  |  | error | 96 | 3059.837 |  |  |

T_0_ (time of germination onset), T_E_ (time at germination capacity) and T_50_ (the time necessary for 50% of viable seeds to germinate).

**Supplementary Table S4.** ANOVA table for seedling traits in the nursery experiment.

|  | **Population variation** | | | | | **Regional variation** | | | | |
| --- | --- | --- | --- | --- | --- | --- | --- | --- | --- | --- |
| **Trait** | **Source of variation** | **d.f.** | **Sum of Squares** | **F Ratio** | **Prob. > F** | **Source of variation** | **d.f.** | **Sum of Squares** | **F Ratio** | **Prob. > F** |
| **shoot length** | population | 23 | 334.15 | 6.85 | **<0.0001** | region | 1 | 64.70 | 12.21 | **0.0008** |
|  | error | 2 | 1.59 | 0.37 | 0.6898 | error | 2 | 0.80 | 0.08 | 0.9275 |
|  |  | 45 | 95.42 |  |  |  | 70 | 371.02 |  |  |
| **root length** | population | 23 | 481.16 | 4.78 | **<0.0001** | region | 1 | 142.36 | 13.45 | **0.0005** |
|  | error | 2 | 36.63 | 4.19 | **0.0215** | error | 2 | 64.78 | 3.06 | 0.0532 |
|  |  | 45 | 196.91 |  |  |  | 70 | 741.12 |  |  |
| **collar diameter** | population | 23 | 10.99 | 5.25 | **<0.0001** | region | 1 | 2.96 | 16.64 | **0.0001** |
|  | error | 2 | 0.04 | 0.22 | 0.8038 | error | 2 | 0.02 | 0.06 | 0.9416 |
|  |  | 45 | 4.09 |  |  |  | 70 | 12.44 |  |  |
| **leaves and shoot mass** | population error | 23 | 1.64 | 3.91 | **<0.0001** | region  error | 1 | 0.56 | 20.23 | **<0.0001** |
|  |  | 2 | 0.00 | 0.12 | 0.8908 |  | 2 | 0.01 | 0.10 | 0.9019 |
|  |  | 45 | 0.82 |  |  |  | 70 | 1.93 |  |  |
| **root mass** | population | 23 | 1.96 | 3.56 | **0.0001** | region | 1 | 0.57 | 15.30 | **0.0002** |
|  | error | 2 | 0.05 | 1.02 | 0.3701 | error | 2 | 0.08 | 1.06 | 0.3527 |
|  |  | 45 | 1.08 |  |  |  | 70 | 2.62 |  |  |
| **total seedling mass** | population | 23 | 6.52 | 3.58 | **0.0001** | region | 1 | 2.26 | 19.46 | **<0.0001** |
|  | error | 2 | 0.05 | 0.30 | 0.7457 | error | 2 | 0.10 | 0.43 | 0.651 |
|  |  | 45 | 3.57 |  |  |  | 70 | 8.14 |  |  |

**Supplementary Table S5.** Pearson correlation coefficient between seed and seedling traits. Bold are significant values (P ≤ 0.05).

|  | seed viability | germination capacity | T_0_ | T_E_ | germination speed | T_50_ | seed. mass | shoot lenght | root. lenght | collar diameter |
| --- | --- | --- | --- | --- | --- | --- | --- | --- | --- | --- |
| germination capacity | 0.340 |  |  |  |  |  |  |  |  |  |
| T_0_ | **0.445** | (-) 0.308 |  |  |  |  |  |  |  |  |
| T_E_ | **0.501** | (-) 0.046 | **0.785** |  |  |  |  |  |  |  |
| germination speed | 0.133 | 0.380 | (-) 0.234 | **0.418** |  |  |  |  |  |  |
| T_50_ | **0.527** | (-) 0.177 | **0.934** | **0.899** | 0.041 |  |  |  |  |  |
| seed.mass | **(-) 0.650** | 0.172 | **(-) 0.545** | (-) 0.378 | 0.206 | **(-) 0.566** |  |  |  |  |
| shoot lenght | (-) 0.191 | **0.431** | **(-) 0.429** | (-) 0.082 | **0.486** | (-) 0.284 | **0.455** |  |  |  |
| root. lenght | 0.038 | 0.260 | (-) 0.276 | (-) 0.050 | 0.316 | (-) 0.205 | 0.392 | **0.502** |  |  |
| collar diameter | (-) 0.304 | 0.337 | **(-) 0.422** | (-) 0.086 | **0.471** | (-) 0.265 | **0.594** | **0.819** | **0.428** |  |
| total seedling mass | (-) 0.140 | **0.490** | (-) 0.346 | (-) 0.158 | 0.255 | (-) 0.266 | **0.554** | **0.682** | **0.633** | **0.661** |

T_0_ (time of germination onset), T_E_ (time at germination capacity) and T_50_ (the time necessary for 50% of viable seeds to germinate).
